# The community health worker model for cardiovascular kidney metabolic syndrome: A new paradigm for high value care

**DOI:** 10.1016/j.ajpc.2025.100944

**Published:** 2025-02-12

**Authors:** Sanjay Rajagopalan, Jeshurun Adarquah-Yiadom, Fonda Mcclain, John Pastor Ansah, Heidi Osborne, Kimberly Nicholson, Zoe Landskroner, Kyia Mountain, Elke Eaton, Jodi Porges, Rita Horvitz, Ian J. Neeland, Peter Pronovost, Robert D. Brook, Jackson T. Wright, Sadeer Al-Kindi, Phillip D. Levy

**Affiliations:** a Harrington Heart and Vascular Institute, University Hospitals, USA; b Case Western Reserve University, School of Medicine, Cleveland, OH, USA; c Better Health Partnerships, Cleveland, OH, USA; d Wayne State University, Detroit, MI, USA; e Houston Methodist Health System, Houston, TX, USA

## Abstract

Access and adherence to prevention and therapeutic lifestyle change programs remain largely aspirational for many low resource and minority communities. Given the importance of prevention and the high cost of care in complex medical conditions such as cardiovascular kidney and metabolic syndrome (CKM), new models of care delivery that enhance value are needed. Community health workers (CHWs) may serve as an innovative link between healthcare systems and the community, improving last mile delivery of services for “at risk” community members through education, outreach, informal counseling, social service support, and advocacy. The impending new Center for Medicare Medicaid Services (CMS) reimbursements for Community Health Integration, Social Determinants of Health (SDOH) assessment, and Principal Illness Navigation services in medically necessary care, represents a major shift in reimbursement models. In this review, we explore four overarching barriers to widespread adoption of CHWs, current roles of CHWs in CKM care, including outcomes and data confirming economic viability and sustainability of engaging CHW's in CKM care. We explore problems with existing financial models for CHW involvement, and forthcoming reimbursement pathways and solutions. CHW's are frontline health workers who could be critical in enhancing value for CKM. However current reimbursement models and restructuring of payments needs to occur rapidly to embrace a new cadre of health workers in our fight against adverse CKM health.

## 1 Introduction

The Cardiovascular Kidney Metabolic (CKM) syndrome is recognized as having substantial origins in social and economic factors that pose barriers to preservation of health [1-3]. While emerging pharmacotherapies to combat CKM syndrome are promising, in a health system burdened by skyrocketing expenditures and a rapidly aging population, alternate approaches are needed. It is increasingly recognized that implementation of existing knowledge can significantly improve outcomes [2]. Access and adherence to many foundational prevention and therapeutic lifestyle change programs remain largely aspirational for many indigent and minority communities[4]. In 2006, the American Public Health Association (APHA) defined CHWs as a trusted members with an unusually close understanding of the community served [5]. This trusting relationship may enable the CHW to serve as a vital intermediary between health/social services and the community, to facilitate access and improve the quality and cultural competence of service delivery. [5,6] CHW's may represent an essential link between bringing cardiometabolic care to “at risk” community members, through a range of activities such as education outreach, informal counseling, social support, and advocacy. [6,7]

## 2 CHWs in cardiometabolic care

CHWs can help obviate multiple barriers in CKM care [6]. First, by identifying and making available local community resources, CHW's can help facilitate access through referrals for various needs including food, housing, and transportation and internet access. Secondly, the cultural understanding that CHWs bring through their own lived experiences may allow empathetic promotion of healthy behaviors such as lowering salt, increasing physical activity and exercise. Thirdly, CHWs can effectively navigate an increasingly complex care delivery model on behalf of their clients, often helping to bridge the “last mile” through consummation of appointments with the right medical professionals and even performing home visits. This is an important problem that is particularly relevant for those with CKM, many of him may have little education or insight into behaviors that facilitate CKM, may suffer from mental disorders, substance abuse or may lack family support systems that prevent successful treatment of CKM syndrome. Finally for many patients, access to evidenced based therapies in CKM remains a substantial issue. By making care personal, and centered on the patient's own life experiences, the CHW helps establish a multidimensional relationship, that is not merely contingent on prescriptive guidelines, but on walking hand in hand with patients to help alleviate fears and anxieties, ultimately improving adherence to care as well as perception of care [7,8].

## 3 Current barriers in incorporating CHWs in cardiometabolic disease

Despite these obvious and persuasive reasons for the incorporation of CHWs, there has been limited adoption, with most traditional payors not reimbursing for CHWs resulting in limited use of CHWs in managing CKM syndrome [6,7,9].

Four important reasons have been cited for the lack of widespread adoption. These include standardized scope of practice for individuals interested in becoming CHWs; lack of knowledge in health systems about the financial impact of CHWs; training of providers on how to utilize CHWs, and finally sustainable financing models for CHWs.

CHWs often have broad roles that creates “scope creep” and a lack of understanding of the boundaries of their role in CKM syndrome [10]. Given the relational nature of their work, CHWs may often perform activities beyond the “official” scope of their job, to meet client needs. The additional expertise to provide guidance in CKM syndrome may require special education [10,11]. Many CHWs currently do not have formal training in medical topics relevant to CKM syndrome. Further, there is still a debate on whether certification is even needed for CHWs as this may place those who become CHWs based on community experience at a disadvantage [7,10].

**Fig. 1:**
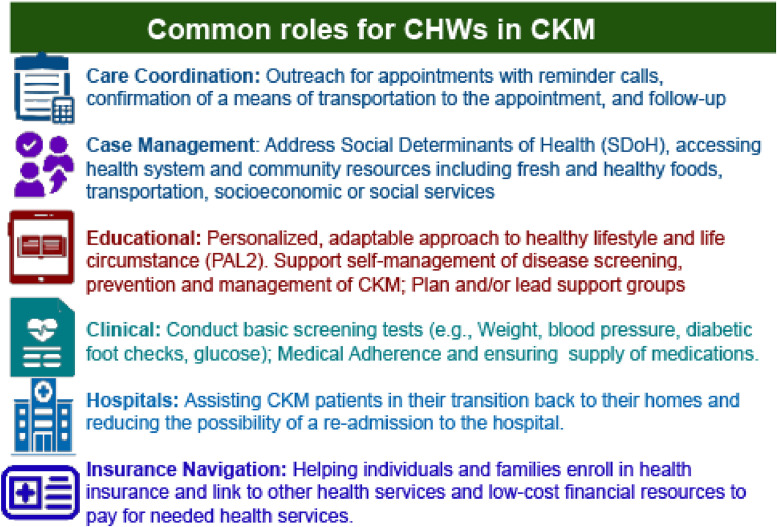
Role for CHWs in CKM Syndrome.

Thus, clarification of essential functions of a CHW for CKM care and narrowing of scope may be essential. CHWs play several roles in diabetes mellitus (DM), hypertension and obesity self-management, including as patient navigators, health promoters, health educators, educating patients on diet, measurement of blood pressure and glucose, healthy lifestyles, addressing social determinants of health, ongoing support including helping monitor vitals at patients’ home, ensuring adherence to cardiometabolic drugs and health system advocacy [7]. Fig. 1 lists common functions that may be expected of a CHW in the care of the cardiometabolic patient [12].

The second most common reason is the lack of health system awareness of the health and economic impact of CHWs in cardiometabolic care. A randomized controlled trial for a CHW diabetes management intervention in Latino populations found A1C levels significantly decreased by 0.42% at 3 months, 0.47% at 6 months, and 0.57% at 12 months. In another study, A1C levels dropped from 10.3% to 7.2% after 3 months, after increasing access to diabetes supplies through CHW efforts[13]. A systematic review of CHW interventions determined they were cost-effective for cardiovascular disease prevention and preventing and managing DM [11]. The median intervention cost per patient per year was $329 for cardiovascular disease (CVD) prevention, $600 for type 2 DM prevention, and $571 for type 2 DM management [11]. Median cost per quality-adjusted life year gained was $17,670 for cardiovascular disease prevention, $17,138 (mean) for type 2 diabetes prevention, and $35,837 for type 2 diabetes management [11]. This may in part be due to the emphasis on lifestyle modification and patient self-management leading to a reduction of avoidable health care [7,14]. CHW intervention has been shown to significantly improve hypertension control, especially in under resourced settings in the United States and across many countries in the world [[15], [16], [17], [18], [19], [20]]. In one of the largest cluster randomized trials (*n* = 2645), CHW intervention when coupled with existing public health infrastructure was effective in driving hypertension control rates at 24 months by 5.2 mm Hg more than placebo (SBP, 95% confidence interval [CI], 3.2 to 7.1; *P* < 0.001). The all-cause mortality was reduced in the intervention arm compared with placebo (2.9% in intervention and 4.3% in the control group) [19]. Evidence for cost reduction and care avoidance can serve as a powerful justification for appropriate hiring and utilization of CHWs [7]. CHWs are very low cost, but highly effective aides in reducing costs for health care systems.^7,9–11,14,21,22^Reduction in health care service utilization, have been demonstrated in a prior meta-analysis, that while acknowledging variability in effect size, demonstrated an important impact in reducing ER visits and hospitalizations [14]. The use of CHWs also resulted in a net savings of $1135 per patient, with each CHW generating a net savings of $170,213 per year [23]. The return on investment (ROI) was 2.3, meaning that for every dollar invested in the CHW program, the hospital saved $2.30 [23].

The third reason for the poor adoption of CHWs is lack of knowledge among providers as how to utilize them in their practice. A key recommendation from a previous report from the Center for Disease Control (CDC), related to training and educating health care providers on the roles that CHWs can play. In this regard, the training of medical students, nurses and allied team members including physicians in utilizing CHWs for the assessment and disposition of social determinants of health needs is already occurring in many different settings but must be accelerated. Provider training is important to creating a supportive environment for integrating CHWs in the health care team.

A final but perhaps overarching reason for the lack of widespread implementation of CHWs has been the lack of stable funding [6,[9], [10], [11],^24^]. In 2014, the Centers for Medicare and Medicaid services (CMS) established a rule expanding preventative services to cover reimbursement for CHW services. However, despite this ruling at the Federal level, there has been substantial variations by state in payments for these services. In a 2022 study, as many as 31 states, did not have a state sanctioned legal mechanism to reimburse CHW services [9]. In many states, although reimbursement for CHWs is enshrined in state law, this does not reliably translate into payment streams. Many legal CHW funding mechanisms have been challenged owing to lack of consistent definition on what constitutes a CHW [9]. Many HCOs are also reluctant to pay the initial costs for a CHW, as the costs are not seen as offsetting the benefits and accrue to diverse players other than the entity funding the initiative. Consequently, it may be challenging for a single CHW employer to provide CHW services free of charge if that person is receiving only a portion of the benefit. Some CHW employers have responded by bundling CHW services with other reimbursable services, but this requires a careful understanding of fund flow and its allocation.

## 4 Current payor models for financing CHWs

In most states a collaborative funding model where grant funding and other pathways including Medicaid managed care organizations are used to support the CHWs’ salary and travel [6,7,9]. In states that have implemented CHW programs, a centralized “hub” model is often utilized [24,25]. In this model, the hub centralizes the services provided by the CHW, and facilitates tactical deployment, as well as uniform service standards and training. Additionally, the hub facilitates payor relationships and appropriate reimbursement pathways [25,26]. This system assists in navigating complex state Medicaid programs. Furthermore, besides ensuring appropriate CHW reimbursement to facilities that employ them, the centralized nature of the HUB allows clinical teams to track the needs of their participant population through client software, facilitating incorporation of local community resources and needs and rapid referral to appropriate services. The HUB model while advantageous, still suffers from the ability to fully fund CHWs. Pathways Community HUB (PCH), contracts with existing organizations or agencies that hire CHWs and provides common data collection tools and pathways. [25,26] A pathway refers to specific goals, typically patient centered and broadly relating to risk factors or defects in health that may translate into an opportunity to mitigate and resolve (Table 1). Each “Pathway” has a unique billing code and modifier assigned to it with an outcome-based unit, which reflects the complexity and time needed to resolve the Pathway [26]. In the PCH model, as much as 50% of payments are directly tied to resolving or “closing” pathways and are passed onto the agency or organization responsible for the CHWs [26]. As the initial investment to hire CHWs must come from the organizations, the idea is that cost recovery from the PCH model would sustain the CHW. However, the extent of reimbursement is tied into contracts between the PCH and the state Medicaid agencies, which vary considerably.

**Table 1:** HUB Pathways Currently Reimbursable.

Developmental Referral	Medical Adherence
Employment	Medical Reconciliation
Family Planning	Medical Screening
Food Security	Mental Health
Health Coverage	Oral Health
Housing	Postpartum
Immunization Referral	Pregnancy
Learning	Social Service Referral
Medical Home	Substance Use
Medical Referral	Transportation

From The Pathways Community HUB Institute Model. Accessible at: https://www.pchi-hub.org/. Accessed on February 20, 2024. .

## 5 Pathways for sustainable models of CHW care

Ideally, a long-term sustainable payor model would necessitate a shift in current funding and reimbursement models, beginning with an alignment of federal and state payment policies to support and incentivize CKM syndrome management. For example, if Medicaid payments were authorized under all state plans for CHW services, under the supervision of a licensed provider, this would create more opportunities for CHWs to work within and outside of hospital systems to improve outcomes. The current disconnects between how CHWs may need to work in CKM and are reimbursed, may need to be addressed. Effective intervention in CKM through CHW's may typically involve multiple domains of care and “opening” as many as 6–8 pathways for each patient. It is unclear how many of these pathways may be reimbursed simultaneously in the same patient (Table 1).

A previous CDC conference provided some concrete suggestions to utilize CHW in DM management, including training in Self-Management, Education Services, social determinants of health related to DM, skills for prompt and early referrals to programs/services by trusted practitioners, training in techniques such as motivational interviewing and other support approaches [10]. These efforts have been largely glucose centric, have lacked the additional focus on hypertension, obesity and kidney disease, and have not integrated technologies that may render these interventions more effective. The integrated use of mobile health (m-health) to mitigate defects in CKM syndrome care including adherence, lifestyle changes, dose titration of drugs are rapidly emerging areas for effective intervention by non-MD providers, particularly in concert with CHWs[27]. There are many examples of m-health interventions utilizing CHWs that have been successful in controlling blood pressure, glycemia and in promoting healthy lifestyle [21,22,28]. With coverage provided for medication adherence and monitoring, additional mechanisms of offsetting the cost of CHWs may be envisioned. The Medicare Shared Savings plan can help offset the cost of initially hiring CHWs, with subsequent savings through preventable health care attainable through the deployment of CHWs representing a sustainable funding mechanism. Financial compensation models for CHWs can be tied to case complexity in patients with CKM. This would greatly be facilitated by mapping CKM stages across health systems or even geographic regions such as county and state geography and assigning resources accordingly. The highest yield will likely be in the high risk (stages 3 and 4) CKM syndrome in order to show financial benefits and cost reduction.

The “Addressing Cardiometabolic Health Inequities by Early PreVEntion in the Great LakEs Region” (ACHIEVE GREATER) is a $18 million-dollar NIMHD funded trial which will evaluate the impact of CHW strategy deployed with a m-health initiative of providing personalized precision health biometrics and a free calcium score initiative for risk assessment in one of its projects in Cleveland, OH. This study partners with the Better Health Partnership, a PCH in Cuyahoga County, that organizes and manages collaborations with various community care agencies that aim to assist vulnerable populations in obtaining access to care and resources [29]. The primary hypothesis of ACHIEVE is that identification and amelioration of SDoH is critical to solve health care deficiencies in CKM care [26,27]. As part of this program, CHWs are specifically trained to provide educational content to patients with risk factors for CKM, through structured interactions and educational events.

CHWs are frontline health workers who are instrumental in connecting people with preventive services and addressing the social determinants of health, especially important to address the rising costs and poor health outcomes seen in CKM syndrome. There is now extensive data suggesting that the use of CHWs is economically viable and sustainable. New ways to incentivize current reimbursement models and restructuring of payments needs to occur rapidly to embrace a new cadre of health workers in our fight against CKM.

## Funding

P50 MD01735 and R35ES031702 from the 10.13039/100000002National Institute of Health.

## Ethical review statement

NA.

## Disclosures

All authors have nothing to disclose.

## Use of generative AI

None used.

## CRediT authorship contribution statement

**Sanjay Rajagopalan:** Writing – review & editing, Writing – original draft, Validation, Supervision, Resources, Project administration, Funding acquisition, Formal analysis, Data curation, Conceptualization. **Jeshurun Adarquah-Yiadom:** Writing – original draft, Methodology, Investigation. **Fonda Mcclain:** Resources, Project administration. **John Pastor Ansah:** Writing – review & editing, Supervision. **Heidi Osborne:** Writing – review & editing. **Kimberly Nicholson:** Project administration. **Zoe Landskroner:** Conceptualization, Methodology. **Kyia Mountain:** Project administration. **Elke Eaton:** Project administration. **Jodi Porges:** Project administration. **Rita Horvitz:** Writing – review & editing. **Ian J. Neeland:** Writing – review & editing, Supervision. **Peter Pronovost:** Writing – review & editing. **Robert D. Brook:** Writing – review & editing. **Jackson T. Wright:** Writing – review & editing. **Sadeer Al-Kindi:** Writing – review & editing, Conceptualization. **Phillip D. Levy:** Writing – review & editing.

## Declaration of competing interest

Funding obtained from National Institutes of Health.
